# Characterization of the lipoxygenase (LOX) gene family in the Chinese white pear (*Pyrus bretschneideri*) and comparison with other members of the Rosaceae

**DOI:** 10.1186/1471-2164-15-444

**Published:** 2014-06-07

**Authors:** Meng Li, Leiting Li, Jim M Dunwell, Xin Qiao, Xing Liu, Shaoling Zhang

**Affiliations:** College of Horticulture, Nanjing Agricultural University, Nanjing, 210095 China; School of Agriculture, Policy and Development, University of Reading, Earley Gate, Reading, UK

**Keywords:** Pear, LOX, Fruit flavor, Gene family, Rosaceae

## Abstract

**Background:**

Lipoxygenases (LOXs), a type of non-haem iron-containing dioxygenase, are ubiquitous enzymes in plants and participate in the formation of fruit aroma which is a very important aspect of fruit quality. Amongst the various aroma volatiles, saturated and unsaturated alcohols and aldehydes provide the characteristic aroma of the fruit. These compounds are formed from unsaturated fatty acids through oxidation, pyrolysis and reduction steps. This biosynthetic pathway involves at least four enzymes, including LOX, the enzyme responsible for lipid oxidation. Although some studies have been conducted on the LOX gene family in several species including *Arabidopsis*, soybean, cucumber and apple, there is no information from pear; and the evolutionary history of this gene family in the Rosaceae is still not resolved.

**Results:**

In this study we identified 107 LOX homologous genes from five Rosaceous species (*Pyrus bretschneideri*, *Malus × domestica*, *Fragaria vesca*, *Prunus mume* and *Prunus persica*); 23 of these sequences were from pear. By using structure analysis, phylogenic analysis and collinearity analysis, we identified variation in gene structure and revealed the phylogenetic evolutionary relationship of this gene family. Expression of certain pear LOX genes during fruit development was verified by analysis of transcriptome data.

**Conclusions:**

23 LOX genes were identified in pear and these genes were found to have undergone a duplication 30–45 MYA; most of these 23 genes are functional. Specific gene duplication was found on chromosome4 in the pear genome. Useful information was provided for future research on the evolutionary history and transgenic research on LOX genes.

**Electronic supplementary material:**

The online version of this article (doi: 10.1186/1471-2164-15-444) contains supplementary material, which is available to authorized users.

## Background

Pear, a member of the subfamily Pomaceae in the Rosaceae, is a temperate fruit crop of major economic importance in the world market. As global economic markets develop, the current breeding objects are moving from the traditional focus on fruit yield to fruit quality, especially flavor. Such flavor is a genetically and biochemically highly complex trait, which involves the interaction of sugars, acids and aroma. Aroma, as a very important component of flavor is receiving more and more attention. The aroma of the fruit is a complex mixture of more than 1000 volatile compounds, including esters, aldehydes, terpenes, alcohols, carbonyl compounds, and some sulfur compounds [1, 2], and the type and relative proportion of each aroma component determine the specific aroma of different fruit. Although intensive research has been conducted on the physiological and biochemical analysis of fruit aroma, the molecular genetic basis and inheritance of aroma patterns are still unknown.

However, biosynthetic pathways leading to the formation of plant flavor volatiles such as esters, alcohols and aldehydes have been thoroughly investigated [3]. As judged by either quality or quantity, the main fruit volatiles are substantially derived from saturated and unsaturated fatty acids. Straight-chain alcohols, aldehydes, ketones, acids, esters and lactones are primarily formed by the fatty acid oxidation pathway by lipoxygenase, via α and β- oxidation. The lipoxygenase pathway of fatty acids involves at least four enzymes, namely lipoxygenase (LOX), hydroperoxide lyase (HPL), alcohol dehydrogenase (ADH) and alcohol acetyl transferase (AAT).

LOXs are lipid-oxidizing enzymes, a type of non-haem iron-containing dioxygenase, which is ubiquitous in the animal and plant kingdoms [4]; they are even found in fungi [5] and bacteria [6]. LOX is a common plant lipoxygenase that oxidizes linoleate and alpha- linolenate, the two most common polyunsaturated fatty acids found in plants. The patterns of LOX gene expression vary according to the tissue and stage of development [7–10].

According to enzyme classification, LOX is defined as a Linoleic: Oxygen oxidoreductase, which catalyzes (Z, Z) -1,4-pentadiene structural units of unsaturated fatty acids plus oxygen to produce unsaturated fatty acid peroxides [11]. It is a multifunctional enzyme, involved in at least three different types of catalytic reaction: 1) oxidation of the lipid double plus (peroxidase reaction); 2) a secondary lipid peroxide conversion (reaction of hydrogen peroxidase) [12]; 3) formation of epoxy leukotrienes (leukotriene synthesis reaction) [13].

It is known that in complex eukaryotes LOXs are generally encoded by a multigene family [10, 14]. With the rapid development in sequencing and functional genomics research, LOX genes are being identified, cloned and studied in more and more plant species.

For example, six lipoxygenases were reported in the model plant *Arabidopsis thaliana*[15]. Studies on cucumber revealed that expression of 13 out of the 23 LOX genes can be detected using RT-PCR. Twelve genes were differentially expressed in response to abiotic stresses and plant growth regulator treatments [16]. Analysis of the grape (*Vitis vinifera* L.) genome revealed that a LOX family consisting of 18 individual members [17]. A shared polyploidy relationship between *Glycine max* and *Medicago truncatula* was revealed by analysis of this gene family [18]. There are three different types of LOX in soybean LOX1, LOX2, and LOX3, while the LOX2 isozyme is primarily responsible for the “beany” flavor of most soybean seeds. A single nucleotide-amplified polymorphism (SNP) marker was found to identify the lack of the LOX2 isozyme, and can be used to assist the breeding and selection in this species [19]. A total of 25 LOX genes were identified in apple by mining the whole assembled apple genome [20].

With the intention to extend the knowledge of the formation of aroma-related volatiles and to understand the structure and evolutionary history of the LOX gene family, we investigated this gene family in pear (*Pyrus bretschneideri*) and compared pear LOXs with those of another four fully sequenced Rosaceae species (*Malus × domestica*, *Fragaria vesca*, *Prunus mume* and *Prunus persica*). Structure analysis, synteny analysis, phylogenetic analysis and positive selection analysis were conducted on LOX homologous genes and the effects on function are discussed. Using the pear genome information, we also utilised the transcriptome dataset of pear fruit to verify our results.

## Results

### Sequence identification and collection

**Figure 1:**
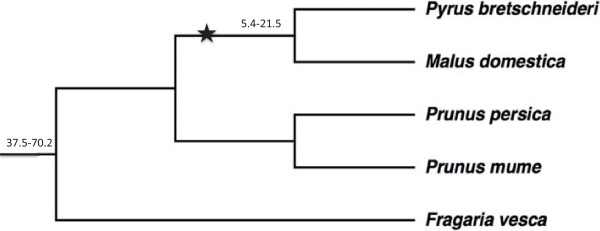
**Species tree of five Rosaceae species.** Star indicates the occurrence of WGD; Numbers in the figures indicate species divergence time. Unit:MYA (million years ago). The data were downloaded from NCBI (http://www.ncbi.nlm.nih.gov/) Common Tree in Taxonomy section and the tree was constructed by MEGA5.

Using the domain of “Lipoxygenase” from Pfam (http://pfam.janelia.org/), we searched for the LOX homologous genes in five fully sequenced genome of Rosaceae species, namely peach (*Prunus persica*) [21], apple (*Malus × domestica*) [22], woodland strawberry (*Fragaria vesca*) [23], mei (*Prunus mume*) [24] and pear (*Pyrus bretschneideri*) [25] (Table 1). By employing the “multiple segment Viterbi” (MSV) algorithm; implemented in HMMER3 software package, 128 sequences were identified. After filtering the length of the homologous genes, 19 genes were removed for shortness and two sequences were removed for not having the complete domain of LOX as tested by SMART (http://smart.embl/heidelberg.de/). Finally, 107 genes were identified as LOX homologous genes (Additional file 1: Table S1).23 LOX genes were identified in pear with 36 in apple, 18 in mei, 16 in peach and 14 in strawberry. The chromosome number of apple and pear is 34 compared with 16 in peach and mei, and 14 in strawberry. The number of LOX genes in pear and apple are almost double the number of in peach and mei. Pear and apple are the most important economic crops in the Rosacease and belong to the Maloideae while mei and peach belong to the Prunoideae, which does not show the expansion of LOX gene family. The species tree of the five Rosaceae species shows the whole genome duplication in the progenitor of pear and apple (Figure 1).

**Table 1:** **The five Rosaceae species in which LOX homologous genes were identified**

Common name	Species name	Chromosome number	Release version	Genome gene number	Identified LOX genes	Gene name prefix
Pear	*Pyrus bretschneideri*	34	NJAU, v1.0	42341	23	Pbr
Apple	*Malus domestica*	34	GDR, v1.0	63541	36	MDP
Peach	*Prunus persica*	16	JGI, v1.0	27864	16	ppa
Mei	*Prunus mume*	16	BFU, v1.0	31390	18	Pm
Woodland strawberry	*Fragaria vesca*	14	GDR, v1.0	32831	14	mrna

In this study we investigated the genome of five Rosaceae species; pear, apple, peach, mei and woodland strawberry. NJAU, Nanjing Agricultural University (http://peargenome.njau.edu.cn/); GDR, Genome Database for Rosaceae (http://www.rosaceae.org/); JGI, Joint Genome Institude (http://www.jgi.doe.gov/); BFU, Beijing Forestry University (http://prunusmumegenome.bjfu.edu.cn/index.jsp).

The pear LOX gene family has 23 members, which are distributed unevenly in the genome. Chromosomes 7, 9 and 10 each have 1 LOX gene while chromosomes 2, 5, 11 and 16 have 2 LOX genes and 4 on scaffold. Most notable is the presence of 8 LOX genes on chromosome 4. We can therefore infer that chromosome 4 has gone through a segmental duplication and the LOX gene family is in that segment.

We also noticed that *Pbr020432.1* (Chr4: 1151461–1156749), and *Pbr020415.1* (Chr4: 1294295–1299591) encode proteins of the same length (Additional file 1: Table S1), with the same pI (Isoelectric Point) and (Molecular Weight). After further analysis, we found out that these two sequences are identical. *Pbr020435.*1 (1121034–1124532) and *Pbr020412.1* (1326550–1330048) on chromosome 4, *Pbr004005.1* (32328–35666) and *Pbr004008.1* (60045–63383) on scaffold1189.0, and *Pbr004541.1* (22844958–22851334) and *Pbr004568.1* (23111942–23118253) on chromsome11 are also identical.

We included all the 25 LOX genes described in the analysis of the LOX gene family in apple [20], with the exception of MDP0000312394, which did not have the complete domain structure.

### Phylogenetic analyses

**Figure 2:**
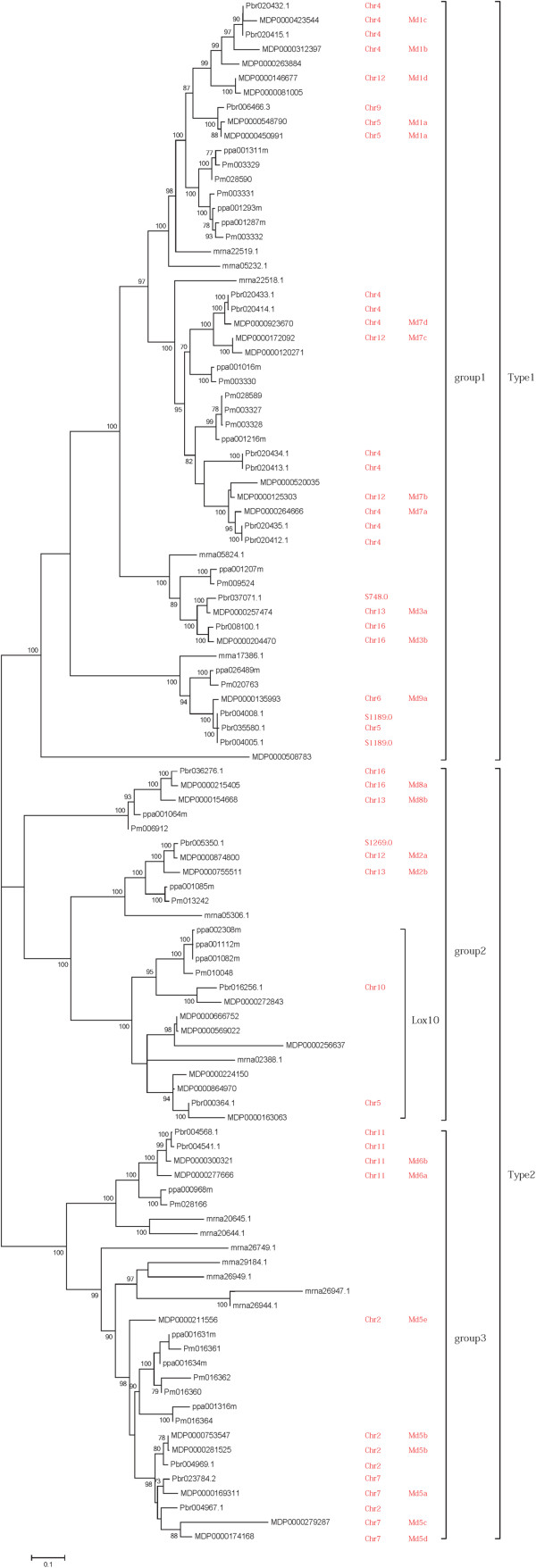
**Phylogenetic tree of LOX homologous genes in five Rosaceae species.** Chr- denotes chromosome and S-denotes scaffold. The phylogenetic tree of 107 genes is shown in this figure. The phylogenetic tree was generated using the neighbor joining (NJ) method in MEGA5. A Bootstrap test was set as 1000 to test the confidence of the tree. The bootstrap values of confidence level, as percentages, are given at branch nodes.

In order to investigate phylogenetic relationships and the molecular evolutionary history of the sequences in these five Rosaceae species (pear, apple, peach, mei and strawberry), a phylogenetic analysis was conducted and a phylogenetic tree was generated using the neighbor joining (NJ) method (Figure 2); the maximum-likelihood (ML) method was also performed in MEGA5. The tree remained consistent using these two methods. A Bootstrap test was set as 1000 to test the confidence of the tree. The NJ tree showed that these 107 sequences clustered into 3 main groups and the ML tree confirmed this result.

We can determine from this phylogeny that the LOX genes were present prior to the evolution of these five species, since all the branches have LOX genes from different species rather than each branch having only the genes from a single species. All the LOX genes fall into two major clades: Type1 and Type2. The first group corresponds to type1 LOX, and the groups 2 and 3 corresponds to type2 LOX. We named the pear LOX genes according to the published studies on apple [20, 26]. The genes were divided into 9 subfamilies while LOX4 is not included in our research since this gene is not really a LOX gene, a conclusion supported by Vogt (2013). However, we identified an additional cluster of LOX genes and named it LOX10.

### Structure and biochemical features of LOX genes

The structure of the LOX gene sequence comprises two domains, the “Lipoxygenase” domain PF00305 in Pfam (http://pfam.xfam.org/), and domain PF01477 which is named PLAT (Polycystin-1, Lipoxygenase, Alpha-Toxin) or LH2 (Lipoxygenase homolog). We identified 107 LOX genes that all the functional domain, PF00305. However, 12 out of 107 did not have the PLAT/LH2 domain which is found in a variety of membrane or lipid associated proteins (Additional file 1: Table S1). All the LOX homologous genes in pear have both domains.

**Figure 3:**
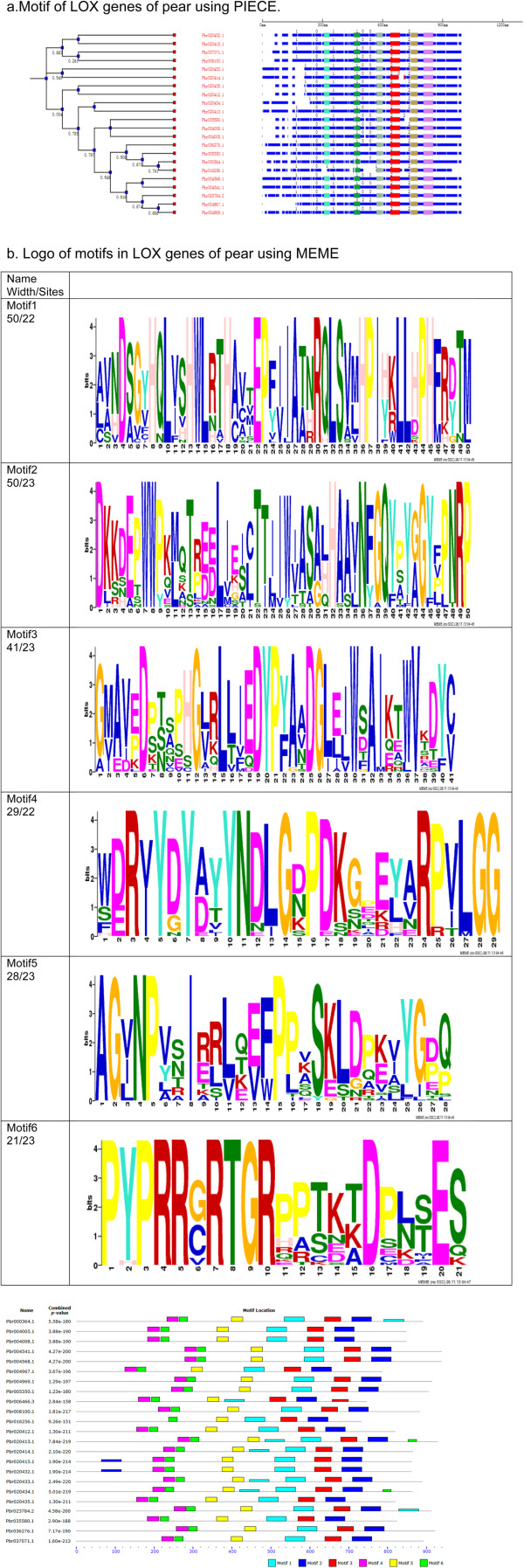
**Structure of LOX genes in pear. a**. Motif of LOX genes using PIECE. 0, 1, 2 denote the phase of the exon. The blue part indicates the exons and different colors indicate different motifs, six motifs are shown in this figure. *Pbr016256.1* has one motif missing, all the others have six motifs. **b**. Logo of motifs in LOX genes of pear using MEME. The line represents the coding sequence and six motifs are shown. All the results were obtained from the amino acid sequence. The total number of pear LOX genes is 23, while the sites means how many in them have this motif and the width is the length of the motif. The Maximum motif width was set as 50 in this study. Pbr016256.1 has one motif missing, all the others have six motifs.

In order to examine the motif structure in pear we constructed a six motif figure by submitting the genomic data and the coding sequence data of LOX genes of pear to the PIECE and MEME website (Figure 3). All the LOX genes have all the six motifs, except Pbr016256.1 which is missing one motif at the beginning of the sequence (Figure 3a). The MEME results (Figure 3b) also confirmed the results. The protein encoded by *Pbr016256.1* has 734 AAs which is the shortest one. However, *Pbr016256.1* is expressed at all the six stages (Table 2) in our transcriptome research, so is a functional gene.

**Table 2:** **Expression of LOX genes at six stages of fruit development in pear. Values shown are RPKM (reads per kilobase per million) values**

GeneID	15 DAF^1^	36 DAF	80 DAF	110 DAF	145 DAF	167 DAF
*Pbr016256.1*	2.2141	2.6069	1.5580	0.3911	0.9660	0.4683
*Pbr004541.1*	1.4712	0.9037	0.3038	1.0372	0.2898	0.1826
*Pbr004568.1*	3.3261	1.4121	0.5469	1.2813	0.0580	0.1826
*Pbr008100.1*	7.4133	14.0537	11.3064	15.8293	0.9860	7.0554
*Pbr036276.1*	53.5461	50.2180	44.8017	42.9360	23.5515	38.6237
*Pbr004967.1*	3.7476	4.4574	10.0988	7.5141	0.4851	2.1109
*Pbr004969.1*	1235.2539	807.6147	1860.9364	1067.5802	190.7420	357.7309
*Pbr020413.1*	-	0.0572	0.1230	-	-	-
*Pbr020414.1*	0.1390	-	0.0660	-	-	-
*Pbr020415.1*	0.1395	0.0616	-	0.0665	-	0.0664
*Pbr020432.1*	0.0697	0.0616	-	0.0665	-	0.0664
*Pbr020433.1*	0.2029	0.2389	0.8352	0.7096	0.1838	0.1931
*Pbr000364.1*	0.0675	-	0.1282	-	-	-
*Pbr035580.1*	0.1457	0.1930	0.0692	-	-	-
*Pbr023784.2*	34.1408	38.0485	241.7718	123.1727	24.9753	69.3049
*Pbr006466.3*	0.6446	0.7827	0.6889	0.5380	-	0.1534
*Pbr005350.1*	106.0468	48.9909	13.1125	17.4077	2.3452	86.6531
*Pbr037071.1*	0.0673	0.1188	0.7027	1.4754	-	-

“-” means expression data are not available. ^1^DAF denotes days after flowering. Of the 23 LOX genes in pear 18 were expressed in pear fruit, while *Pbr004005.1*, *Pbr004008.1*, *Pbr020412.1*, *Pbr020434.1* and *Pbr020435.1* were not expressed; 10 of these18 genes were expressed at all six stages.

### Collinearity analyses

We found 72 paired collinearity relationships from these 107 sequences, of which 58 pairs are collinear between pear and other species (Table 3). All the collinearity relationships are formed by WGD or segmental duplication according to the result of MCscan. This corresponds to the chromosome collinearity between pear and apple.

**Table 3:** **Collinearity relationship of pear LOX genes to the other four species**

Synteny sequence1	Chromosome	Synteny sequence2	Chromosome
*Pbr004967.1*	Chr2	*Pm016360*	Chr5
*Pbr004967.1*	Chr2	*ppa001316m*	Chr2
*Pbr004967.1*	Chr2	*mrna26944.1*	Chr7
*Pbr020414.1*	Chr4	*Pm003327*	Chr1
*Pbr020414.1*	Chr4	*ppa001216m*	Chr6
*Pbr020414.1*	Chr4	*MDP0000172092*	Chr12
*Pbr020414.1*	Chr4	*MDP0000264666*	Chr4
*Pbr020415.1*	Chr4	*Pm003329*	Chr1
*Pbr020415.1*	Chr4	*ppa001311m*	Chr6
*Pbr020415.1*	Chr4	*MDP0000146677*	Chr12
*Pbr020415.1*	Chr4	*MDP0000923670*	Chr4
*Pbr020415.1*	Chr4	*mrna22518.1*	Chr6
*Pbr020432.1*	Chr4	*Pm003329*	Chr1
*Pbr020432.1*	Chr4	*ppa001311m*	Chr6
*Pbr020433.1*	Chr4	*MDP0000146677*	Chr12
*Pbr020433.1*	Chr4	*mrna22518.1*	Chr6
*Pbr020434.1*	Chr4	*MDP0000312397*	Chr4
*Pbr020435.1*	Chr4	*Pm003327*	Chr1
*Pbr020435.1*	Chr4	*ppa001216m*	Chr6
*Pbr020435.1*	Chr4	*MDP0000172092*	Chr12
*Pbr020435.1*	Chr4	*MDP0000264666*	Chr4
*Pbr000364.1*	Chr5	*ppa001082m*	Chr4
*Pbr000364.1*	Chr5	*MDP0000224150*	Chr5
*Pbr000364.1*	Chr5	*MDP0000272843*	Chr10
*Pbr000364.1*	Chr5	*mrna02388.1*	Chr3
*Pbr035580.1*	Chr5	*Pm020763*	Chr6
*Pbr035580.1*	Chr5	*ppa026489m*	Chr8
*Pbr035580.1*	Chr5	*MDP0000135993*	Chr6
*Pbr035580.1*	Chr5	*mrna17386.1*	Chr2
*Pbr023784.2*	Chr7	*Pm016360*	Chr5
*Pbr023784.2*	Chr7	*ppa001316m*	Chr2
*Pbr023784.2*	Chr7	*MDP0000174168*	Chr7
*Pbr023784.2*	Chr7	*MDP0000281525*	Chr2
*Pbr023784.2*	Chr7	*mrna26944.1*	Chr7
*Pbr006466.3*	Chr9	*MDP0000450991*	Chr9
*Pbr016256.1*	Chr10	*ppa001082m*	Chr4
*Pbr016256.1*	Chr10	*MDP0000224150*	Chr5
*Pbr016256.1*	Chr10	*MDP0000272843*	Chr10
*Pbr004541.1*	Chr11	*Pm028166*	scaffold103
*Pbr004541.1*	Chr11	*ppa000968m*	Chr6
*Pbr004541.1*	Chr11	*MDP0000277666*	Chr11
*Pbr004541.1*	Chr11	*mrna20644.1*	Chr3
*Pbr004568.1*	Chr11	*Pm028166*	scaffold103
*Pbr004568.1*	Chr11	*ppa000968m*	Chr6
*Pbr004568.1*	Chr11	*MDP0000277666*	Chr11
*Pbr004568.1*	Chr11	*mrna20644.1*	Chr3
*Pbr008100.1*	Chr16	*Pm009524*	Chr2
*Pbr008100.1*	Chr16	*ppa001207m*	Chr1
*Pbr008100.1*	Chr16	*MDP0000204470*	Chr16
*Pbr008100.1*	Chr16	*MDP0000257474*	Chr13
*Pbr008100.1*	Chr16	*mrna05824.1*	Chr4
*Pbr036276.1*	Chr16	*Pm006912*	Chr2
*Pbr036276.1*	Chr16	*ppa001064m*	Chr1
*Pbr036276.1*	Chr16	*MDP0000154668*	Chr13
*Pbr004005.1*	scaffold1189.0	*Pm020763*	Chr6
*Pbr004005.1*	scaffold1189.0	*MDP0000135993*	Chr6
*Pbr037071.1*	scaffold748.0	*Pm009524*	Chr2
*Pbr037071.1*	scaffold748.0	*ppa001207m*	Chr1

We found 72 paired collinear relationships from these 107 sequences, of which 58 pairs are collinear between pear and other species. All the collinearity relationships are due to WGD or segmental duplication according to the result of MCscan. *Pbr004541.1*, *Pbr004568.1* and *Pbr020415.1*: these three genes in pear have a collinear relationship with LOX genes in all the other four species.

**Figure 4:**
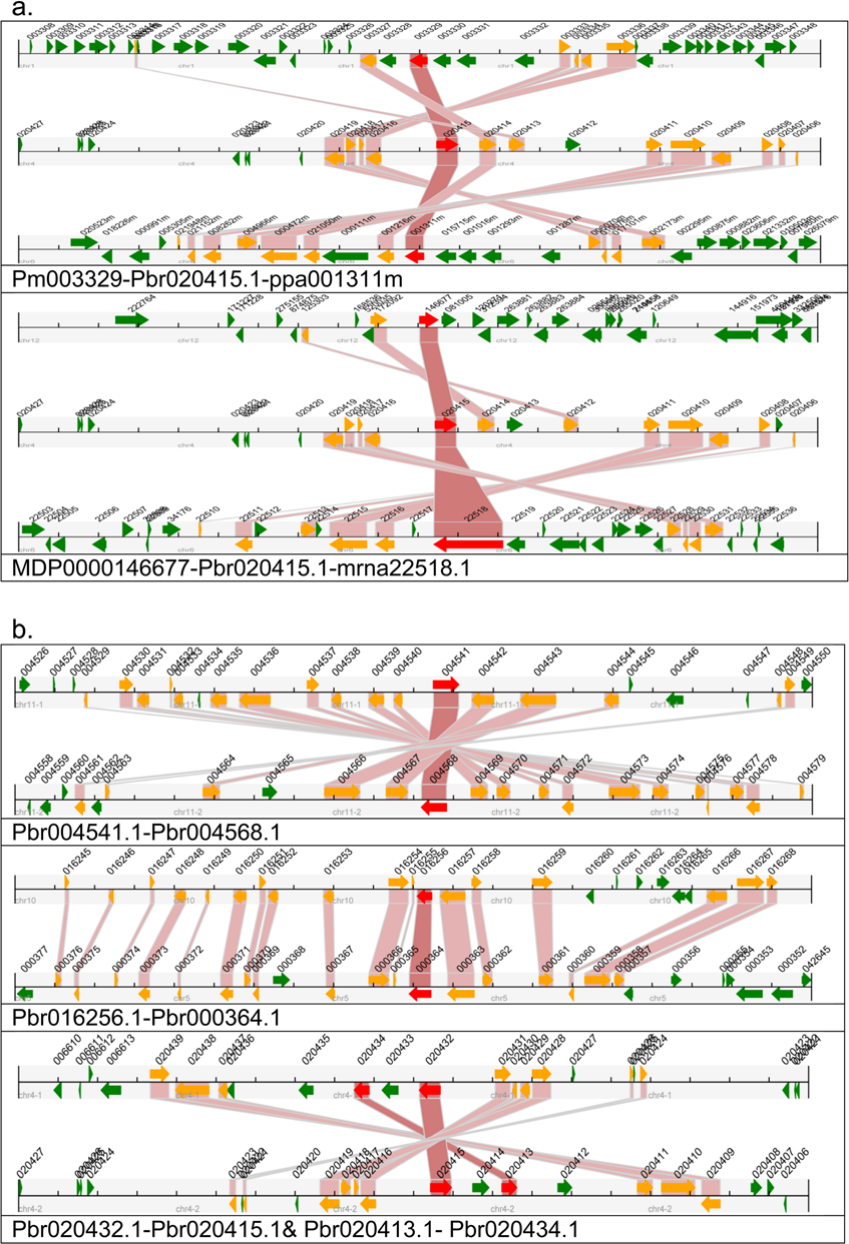
**Collinearity relationships of LOX genes in pear.** A region of 100 kb on each side flanking the LOX genes (the red one) is shown in this figure. Homologous gene pairs are connected with bands. The genes on the scaffold are not included. The black horizontal scale line represents the chromosome segment with the chromosome name under the line, and the broad line with arrowhead represents genes and its transcriptional orientation. The gene names suffix is the text besides the line. The LOX genes are shown in red, homologous genes are shown in yellow and other genes shown in green. Pbr- denotes a pear gene, MDP- apple, ppa- peach, Pm- Mei and mrna- strawberry. **a**. The example of *Pbr020415.1* to show the synteny relationship among different species. **b**. The collinearity relationships of LOX genes in pear.

The three pear genes *Pbr004541.1*, *Pbr004568.1* and *Pbr020415.1* have a collinearity relationship with LOX genes in all the other 4 species used in this analysis. We used *Pbr020415.1* as an example to show the collinearity relationship of different species (Figure 4a).

Collinearity relationships were also found among the same species (Table 4). 7 pairs are among pear, 6 pairs are among the apple and 1 in strawberry. Pbr004005.1 on scaffold1189.0 is collinear with Pbr004008.1 and Pbr035580.1. All the 4 pairs of LOX paralogous genes in pear were shown to have a collinear relationship. An illustration of the collinearity relationship of LOX genes on the pear chromosome is given in (Figure 4b). This collinearity occurs in either the sense or antisense direction.

**Table 4:** **The collinearity relationship among LOX genes in the same species**

Synteny sequence1	Chromosome	Synteny sequence2	Chromosome
*mrna20644.1*	Chr3	*mrna26944.1*	Chr7
*Pbr020413.1*	Chr4	*Pbr020434.1*	Chr4
*Pbr020415.1*	Chr4	*Pbr020432.1*	Chr4
*Pbr000364.1*	Chr5	*Pbr016256.1*	Chr10
*MDP0000279287*	Chr7	*MDP0000281525*	Chr2
*MDP0000272843*	Chr10	*MDP0000569022*	Chr5
*Pbr004541.1*	Chr11	*Pbr004568.1*	Chr11
*MDP0000081005*	Chr12	*MDP0000423544*	Chr4
*MDP0000125303*	Chr12	*MDP0000264666*	Chr4
*MDP0000146677*	Chr12	*MDP0000923670*	Chr4
*MDP0000154668*	Chr13	*MDP0000215405*	Chr16
*Pbr008100.1*	Chr16	*Pbr037071.1*	scaffold748.0
*Pbr004005.1*	scaffold1189.0	*Pbr004008.1*	scaffold1189.0
*Pbr004005.1*	scaffold1189.0	*Pbr035580.1*	Chr5

In total 14 pairs of collinear relationships were detected among the same species. 7 pairs are among pear, 6 pairs are among apple and 1 in strawberry. *Pbr004005.1* on scaffold1189.0 is collinear with *Pbr004008.1* and *Pbr035580.1*. All the 4 pairs of LOX paralogous genes in pear are included in the collinearity analysis.

### Expression of LOX genes in pear

**Figure 5:**
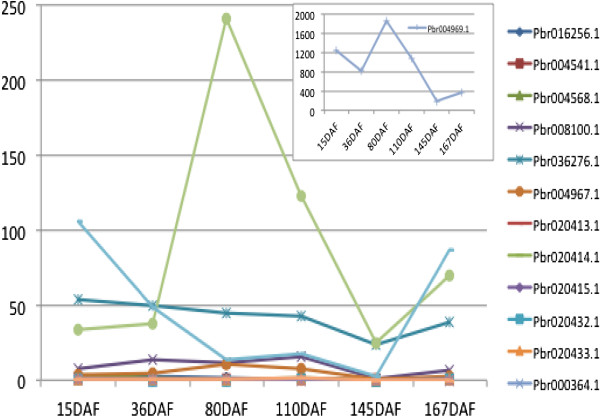
**Expression level of LOX genes in pear fruit.** The value on the y-axis is the RPKM (reads per kilobase per million) value for each gene. The x-axis represents the six stages of pear fruit development, 15 days after flowering (DAF), 36 DAF, 80 DAF, 110 DAF, 145 DAF, and 167DAF.

We sequenced RNA from mixed pear fruit samples to obtain transcriptome data from six stages of fruit development (15 days after flowering (DAF), 36DAF, 80DAF, 110DAF, 145DAF, 167DAF) (Table 2). Of the 23 LOX homologous genes identified in pear, 18 were found to be expressed in the fruit, while the *Pbr004005.1*, *Pbr004008.1*, *Pbr020412.1*, *Pbr020434.1* and *Pbr020435.1* are not expressed; 10 of the 18 were found to be expressed in all the six stages. The average expression RPKM (reads per kilobase per million) value of all the pear genes is 35.17 while the average RPKM value of the LOX gene family is 77.82 which is double the average value. Therefore, most LOX genes are functionally active.We also examined the expression level figure of these genes (Figure 5). The results showed that the expression of LOX genes in pear was usually low expression in the early development stage (15 DAF), increased to a peak at the middle development stage (80 DAF or 110 DAF), reduced to near zero near ripening (145 DAF) and then increased slightly at ripening (167 DAF). This is believed to correspond to the pattern of changes in the volatile components of pear fruit aroma.

## Discussion

The LOX gene family is involved in the development of various plant organs, in the fruit ripening process also in the response to abiotic stresses, and is also involved in the synthesis of aldehydes and alcohols. Most importantly, it plays a key role at the early stage in the synthetic pathway for fruit aroma compounds and therefore the development of fruit flavor.

By using the MSV algorithm in HMMER3, we searched the whole genome sequences of five Rosaceae species; a total of 107 LOX homologous genes were identified. The LOX gene family exists before the evolutionary distinction of these five species. Phylogenetic analysis, structure analysis, collinearity analysis, and transcriptome expression analysis were conducted on all the 107 LOX genes. In summary, this study provides a characterization of LOX gene family in pear and the evolutionary history of LOX in Rosaceae.

36 LOX genes were identified in apple and 23 genes in pear, 18 in mei, 16 in peach and 14 in woodland strawberry. The number of LOX genes in pear and apple was almost double the number in mei, peach and strawberry. In pear we identified 23 LOX genes, which is a comparatively large gene family. The LOX gene family has gone through an expansion which corresponds to the variation in chromosome number. For example, the chromosome number of apple and pear is 34 compared to 16 in peach and mei, and 14 in strawberry. Considering a recent WGD (whole genome duplication) event was shared by apple and pear at 30-45MYA (million years ago) (Figure 1) [25], LOX gene family expansion in apple and pear is believed to have occurred at that time.

From the observation of the phylogenetic tree (Figure 2), the LOX genes of pear from the same chromosome (chromosome 4 and chromosome 11) cluster together, which indicates the similarity of the genes on the same chromosome. Also the duplicated copies on chromosome 4 (*Pbr020432.1* - *Pbr020415.1*, *Pbr020435.1* - *Pbr020412.1*), chromosome 11 (*Pbr004541.1* - *Pbr004568.1*) and scaffold1189.0 (*Pbr004005.1* - *Pbr004008.1*) of the pear genome are paralogous genes. The two pairs of LOX genes located on chromosome 4 (Additional file 1: Table S1) have the same sequence and their order on the chromosome is the same. All these 4 pairs of genes are distributed on the same chromosome or scaffold and from comparison of their position on the chromosome it can be concluded that tandem duplication or segmental duplication has happened in this area of chromosome. Pear LOX genes showed an obvious trend of expansion with duplication as the most common occurrence. It can be concluded that a tandem duplication or segmental duplication has happened in the pear genome. However, the specific type of duplication is still unknown.

In addition, transcriptome data showed that 18 of the LOX genes in pear were expressed. This confirms that these genes are functionally active, with 10 of them being expressed in all six stages of fruit development. Though one motif was missing, gene *Pbr016256.1* is still expressed in our study at all the six stages; this suggests that the loss of this N-terminal domain does not affect its function. The change in expression level shown in Figure 5 is believed to correspond to the change in aroma volatile components in the fruit. The aroma is very low at the early stage of fruit development and then increases significantly during the middle stage of development. Then subsequently the aroma substances are converted to volatile components. Fruit immediately prior to and during ripening and ripening time have a low level of LOX expression but because of the accumulation of precursors at these development stages the fruit keep emitting an aroma.

Collinearity analyses showed that 331 blocks and 9257 gene pairs have a collinear relationship between pear and apple. Also the chromosomes in pear are collinear to each other, a conclusion confirmed by the pear genome sequencing project, which demonstrated strong collinearity between segments of several chromosomes. In addition, we discovered that a segment in chromosome 4 of the pear genome is believed to be collinear with chromosome 6 in the peach genome. The LOX gene family members *Pbr020432.1* and *Pbr020415.1* on chromosome 4 of pear are collinear with ppa001311m on chromosome 6 of peach; and *Pbr020414.1* and *Pbr020435.1* on chromosome 4 of pear are collinear with *ppa001216m* on chromosome 6 of peach. All these data indicated the collinear relationship of these two chromosomes.

The present analysis included all the LOX genes previously described in apple [20]; and only one was not analysed because it lacked the complete domain structure. We obtained identical phylogenetic results as those in the previous analysis in apple. Furthermore, we also identified a new cluster of LOX genes which were not described in the previous study.

Currently, research studies on fruit volatile substances are focused on the analytical determination of volatile aromatic substances and on how the various cultivation practices and storage conditions affect the aroma of the fruit. Most of this research is focused on the European pear (*Pyrus communis*), with few reports on the Asiatic pear. There is still little knowledge of the molecular mechanism of the volatile aromatic substances. However in this paper we used the material of the Asiatic pear genome of *Pyrus bretschneideri* which is the first pear genome to be sequenced.

We aimed to study the variation in gene sequence structure and to analyse the phylogenic evolutionary relationship of the LOX gene family in the Rosaceae. This information can be used to guide future research on the evolutionary history on LOXs and associated transgenic research. This study of LOX genes is one part of the whole analysis of aroma of pear fruit. Future work will be conducted on the study of the identification of genes of the aroma pathway and the interaction network between these genes.

## Conclusions

107 genes were identified as LOX homologous genes from five Rosaceae species; 23 of these sequences were from pear. Specific gene duplication was found on chromosome4 in the pear genome. All the LOX genes fall into three groups according to phylogenetic analysis. The first group corresponds to type1 LOX, and the groups 2 and 3 corresponds to type2 LOX. Of the 23 LOX homologous genes identified in pear, 18 were found to be expressed in the fruit. The express pattern in six stages was found to correspond to the pattern of changes in the volatile components of pear fruit aroma. The research on the LOX gene family could lead to a better understanding of the molecular mechanism of aroma in fruit. Moreover, the findings of this study may facilitate the research on evolutionary history and transgenic research on LOX genes and other aroma genes.

## Methods

### Sequence identification and collection

By searching “Lipoxygenase” in Pfam (http://pfam.janelia.org/), seeds of Lipoxygenase domain PF00305 were downloaded to our local server. By employing the “multiple segment Viterbi” (MSV) algorithm; implemented in HMMER3 software package [27], we searched for the LOX homologous genes in fully sequenced genome of five Rosaceae species, namely peach (*Prunus persica*), apple (*Malus × domestica*), woodland strawberry (*Fragaria vesca*), mei (*Prunus mume*) and pear (*Pyrus bretschneideri*). 128 genes were obtained with an E value < 1e-10. After filtering according to length, 19 sequences were removed due to their shortness, and two sequences were removed for not having the LOX domain tested by SMART (http://smart.embl/heidelberg.de/). Finally 107 LOX homologous genes were identified in this study.

### Phylogenic analysis

Coding sequence alignment was performed using MUSCLE (Multiple Sequence Caparison by Log-Expectation) with default parameters in Molecular Evolutionary Genetics Analysis-MEGA5 [28]. The neighbor joining trees were constructed with bootstrap 1000 using MEGA5. Maximum-likelihood trees with bootstrap 1000 were also generated using MEGA5 with default settings.

The tree of the five Rosaceae species was obtained by downloading data from NCBI (http://www.ncbi.nlm.nih.gov/): Common Tree in the Taxonomy section. The tree was constructed using MEGA5.

### Motif and analysis of the LOX genes and proteins

Motif analysis was conducted on the website Plant Intron Exon Comparison and Evolution database (PIECE, http://wheat.pw.usda.gov/piece/FAQ.php) [29]. Coding sequences against the genomic sequence were used to plot the figure with motif number six. Based on the Pfam motif, a phylogenetic tree was reconstructed for each gene category by integrating exon-intron and protein motif information. We also combined this database with the MEME (http://meme.nbcr.net) web servers to draw motifs of the sequences and the information of the motifs was collected.

Basic data about the LOX proteins was calculated as follows: amino acid number (aa number); molecular weight (MW) and isoelectric point (pI).

### Collinearity analysis

The whole genome sequences of the five Rosaceae species were downloaded to our local server. Then MCscan (Multiple Collinearity Scan) [30] was used to obtain the collinearity relation of each pair of species. The resulting collinearity chains were evaluated using a procedure in ColinearScan and an E-value < 1e-10 was used as the cutoff.

### Transcriptome sequencing

To examine the expression of pear genes, pear (*Pyrus bretschneideri*) fruit samples at 15 d, 36 d, 80 d, 110 d, 145 d and 167 d after flowering (DAF) were used. Three or more fruits were collected at each stage from the Nanjing Agricultural University experimental farm in 2011. Fruits of the same stage were combined and total RNA was extracted for RNA sequencing. RNA sequencing libraries were constructed using an Illumina standard mRNA-Seq Prep Kit (TruSeq RNA and DNA Sample Preparation Kits version 2). During the production of sequencing libraries, small RNAs ligated with adaptors were used to run RT-PCR. After that, the products were purified and sequenced on an Illumina Hi-seq 2000 Sequencer.

### Availability of supporting data

The data sets supporting the results of this article are included within the article (and its additional files) and raw RNA-seq reads available in the National Center for Biotechnology Information repository under accession PRJNA185970 (http://www.ncbi.nlm.nih.gov/bioproject/PRJNA185970).

## Electronic supplementary material

Additional file 1: Table S1: Structural and biochemical information of LOX homologous genes in five Rosaceae species. aa: amino acid; MW: molecule weight; pI: isoelectric point. ‘0’ means gene has no PLAT/LH2 domain and ‘1’ means the gene has PLAT/LH2 domain. LOX domain is the same. Pbr- denotes a pear gene, MDP- apple, ppa- peach, Pm- Mei and mrna- strawberry. LOX domain is PF00305 in Pfam (http://pfam.xfam.org/), and domain PF01477 is PLAT (Polycystin-1, Lipoxygenase, Alpha-Toxin) or LH2 (Lipoxygenase homology). 23 genes were identified in pear, 36 in apple; 18 in mei, 16 in peach, and 14 in strawberry. (XLSX 17 KB)
